# Fiber Bragg Grating Sensors toward Structural Health Monitoring in Composite Materials: Challenges and Solutions

**DOI:** 10.3390/s140407394

**Published:** 2014-04-23

**Authors:** Damien Kinet, Patrice Mégret, Keith W. Goossen, Liang Qiu, Dirk Heider, Christophe Caucheteur

**Affiliations:** 1 Electromagnetism and Telecommunication Department, Faculty of Engineering, University of Mons, Boulevard Dolez 31, 7000 Mons, Belgium; E-Mails: Damien.kinet@umons.ac.be (D.K.); patrice.megret@umons.ac.be (P.M.); 2 Department of Electrical and Computer Engineering, Evans Hall Newark 202, University of Delaware, Newark, DE 19716-3130, USA; E-Mails: goossen@ece.udel.edu (K.W.G.); heider@udel.edu (D.H.); 3 Source Photonics, 20550 Nordhoff Street, Chatsworth, CA 91311, USA; E-Mail: liangqchiu@gmail.com

**Keywords:** composite materials, sensors, optical fibers, fiber Bragg gratings, structural health monitoring, temperature, strain, interrogation techniques, connectors

## Abstract

Nowadays, smart composite materials embed miniaturized sensors for structural health monitoring (SHM) in order to mitigate the risk of failure due to an overload or to unwanted inhomogeneity resulting from the fabrication process. Optical fiber sensors, and more particularly fiber Bragg grating (FBG) sensors, outperform traditional sensor technologies, as they are lightweight, small in size and offer convenient multiplexing capabilities with remote operation. They have thus been extensively associated to composite materials to study their behavior for further SHM purposes. This paper reviews the main challenges arising from the use of FBGs in composite materials. The focus will be made on issues related to temperature-strain discrimination, demodulation of the amplitude spectrum during and after the curing process as well as connection between the embedded optical fibers and the surroundings. The main strategies developed in each of these three topics will be summarized and compared, demonstrating the large progress that has been made in this field in the past few years.

## 1. Introduction

Composite materials are obtained by assembling at least two non-miscible (but presenting a high adhesion capability) constituents that confer to the assembly physical properties not present in the constitutive materials taken alone [1]. Composite materials are most often made by association of reinforcing fibers with a matrix (thermoplastic or heat-hardening resin). Reinforcing fibers allow enhancing mechanical properties (mechanical resistance, rigidity, hardness, …) and physical properties (thermal resistance, fire resistance, electric properties, …). They have a low density and are easy to handle. There exist different forms (short or continuous fibers) as well as different types. The most often encountered are glass fibers and carbon fibers. The matrix is present to guarantee a proper transfer of the strain to the reinforcing fibers, to protect them against external perturbations and to ensure, after molding, the geometrical form of the structures. The matrix has to be compatible to the reinforcing fibers while ensuring some deformability. It is generally a thermoplastic or thermoset resin.

Composite materials can be built by different techniques. Of course, the most straightforward is molding, which can be performed simply through contact (reinforcing fibers placed in the mold are manually impregnated with the resin), under vacuum or with the more advanced resin transfer molding (RTM) process. Exhaustive details on the manufacturing techniques can be found in [2].

Nowadays, newly produced materials are more and more smart in the sense that they embed sensors and/or metrological tools able to provide real time information about the internal stress state. As such, they intrinsically ensure predictive maintenance. This feature is highly appreciated as it can mitigate the risk of failure due to a sudden breakage, on the one hand, and decrease the maintenance costs, on the other hand. Of course, smart composite materials are also envisioned. Considering the composition and geometry of composite materials, optical fiber sensors are very well suited to be embedded into composite materials without altering their mechanical performances.

Optical fibers are cylindrical silica waveguides made of two concentric layers, the core and the cladding, which guide light thanks to a slight refractive index difference between both layers [3]. Fibered sensors most often exploit telecommunication-grade single-mode optical fibers made of a ∼8 microns thick core in silica doped with germanium oxide surrounded by a 125 microns thick cladding in pure silica. The attenuation is minimum at the wavelength 1,550 nm and is equal to 0.2 dB/km. Naturally, this wavelength range is also exploited for sensing purposes. Optical fiber sensors present decisive benefits [4]. In terms of reliability, they are passive devices and are characterized by a long life time (more than 20 years). They are stable over time (no calibration required) and do not suffer from corrosion. Cables and connectors are telecommunication grade. In terms of performances, they are compatible with multiplexing, offering (quasi-)distributed measurement capabilities. Hence, several tens of sensing points can be cascaded along a single optical fiber. The use of light ensures their immunity against electromagnetic radiation and their insensitivity to radio frequency interference. Also, there is no risk of high voltage discharge and they are explosion safe. Finally, their lightweight and small dimensions allow them to be easily embedded.

Among the different optical fiber configurations, fiber Bragg gratings (FBGs) photo-inscribed in the core of an optical fiber are the most widespread for use in composite materials [5]. They correspond to a refractive index modulation of the fiber core along the fiber axis and behave as selective mirrors in wavelength. They are intrinsically sensitive to temperature, pressure and axial strain and yield a wavelength-encoded response, which can be straightforwardly recorded and processed [6].

In practice, as they have to correctly sustain the fabrication process of composite materials, their integration and their subsequent proper operation require some know-how, as issues at different levels have to be solved. Reference [7] is a recent review about strain measurements in composite laminates with FBG sensors. It provides numerous references to both uni-axial and multi-axial strain sensing applications and to temperature compensation methods. The present review complements well the one of [7] by bringing up-to-date references and by addressing two other relevant issues related the use of FBG strain sensors embedded into composite materials. After a brief introductory part about the operating principle of FBGs, this review article will focus on three specific challenges. Section 3 will address the experiments made to ensure temperature-insensitive strain measurements or simultaneous temperature and strain measurements. The main demodulation techniques used to record and process the amplitude spectrum during and after the curing process will then be overviewed in Section 4. Finally, Section 5 will concentrate on issues associated with the connection between the embedded optical fibers and the surroundings. For each of these three topics, the main solutions will be reviewed while their relative performances will be compared. Other concerns related to the distortion of the composite materials in the surroundings of the optical fibers, on the one hand, and arising from multi-axial strain sensing, on the other hand, will not be addressed here. The reader is invited to consult references [7–9] for details about these aspects.

## 2. Fundamentals on FBGs

An FBG is a periodic and permanent modification of the core refractive index value along the optical fiber axis [5,10]. This modification is usually obtained by transversally exposing the core of a photosensitive optical fiber to an intense interference pattern of ultraviolet light at a wavelength around 240 nm. Indeed, due to the presence of germanium oxide dopants inside the core, an optical fiber is photosensitive (*i.e.*, it benefits from the property to permanently change its refractive index when exposed to light) in a wavelength band centered around 240 nm. For this reason, continuous-wave frequency-doubled argon-ion laser emitting at 244 nm or pulsed excimer laser emitting at 248 nm are most often used to manufacture FBGs. To create an interference pattern, two writing techniques are privileged: the interferometric (or transverse holographic) method and the phase mask technique.

**Figure 1.**
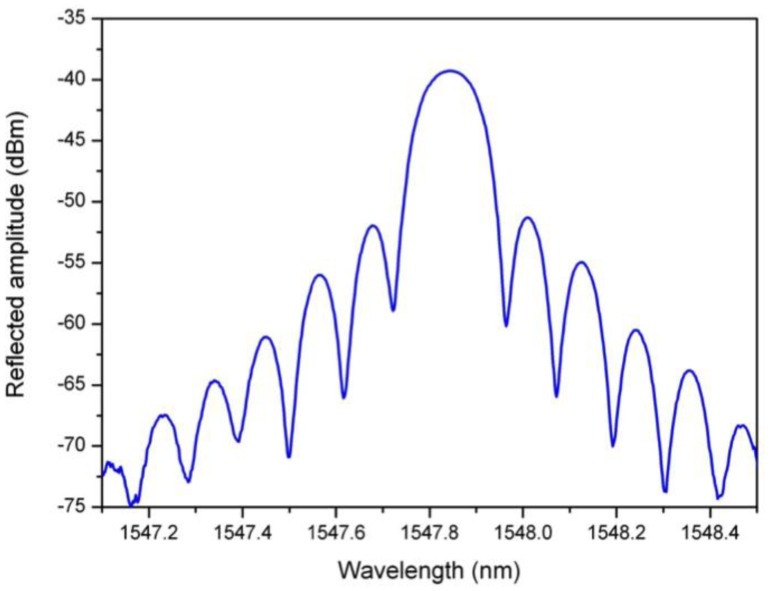
Reflected amplitude spectrum of a 1 cm long uniform FBG.

An FBG is defined by several physical parameters. The grating length L is the optical fiber length along which the refractive index modulation is realized. The periodicity and the amplitude of the refractive index modulation are labeled Λ and δn, respectively. The order of magnitude of these parameters typically varies between 200 nm and 1,000 nm for Λ, from a few mm to a few tens of cm for L and from 10^−5^ to 10^−3^ for δn. Such a perturbation induces light coupling between two counter-propagating core modes. This mode coupling occurs for some wavelengths around the Bragg wavelength defined by:

(1) $$\lambda_{\text{Bragg}}=2n_{\text{eff}}\Lambda$$

where *n*_eff_ is the effective refractive index of the core mode at the Bragg wavelength. A uniform FBG acts as a selective mirror in wavelength around the Bragg wavelength to yield a pass-band reflected amplitude spectrum, as depicted in Figure 1 for a 1 cm long FBG. In fact, at each refractive index discontinuity along the fiber axis, a weak Fresnel reflection is generated. They add in phase at the Bragg wavelength, yielding an important reflection band surrounded by side lobes.

In practice, the effective refractive index of the core and the spatial periodicity of the grating are both affected by changes in strain and temperature. In particular, the effective refractive index is modified through the thermo-optic and strain-optic effects, respectively. Hence, from Equation (1), the Bragg wavelength shift Δλ_B_ due to strain Δ*ε* and temperature Δ*T* variations is given by:

(2) $$\Delta \lambda_{\text{B}}=2\left(\Lambda \frac{\text{d}n_{\text{eff}}}{\text{d}T}+n_{\text{eff}}\frac{\text{d}\Lambda }{\text{d}T}\right)\Delta T+2\left(\Lambda \frac{\text{d}n_{\text{eff}}}{\text{d}\varepsilon }+n_{\text{eff}}\frac{\text{d}\Lambda }{\text{d}\varepsilon }\right)\Delta \varepsilon$$

The first term in Equation (2) represents the effect of temperature on the Bragg wavelength. The Bragg wavelength shift due to thermal expansion comes from the modification of the grating spacing and the refractive index. The relative wavelength shift due to a temperature change Δ*T* can be written as:

(3) $$\frac{\Delta \lambda_{\text{B}}}{\Delta T}=\lambda_{\text{B}}\left(\frac{1}{n_{\text{eff}}}\frac{\text{d}n_{\text{eff}}}{\text{d}T}+\frac{1}{\Lambda }\frac{\text{d}\Lambda }{\text{d}T}\right)$$

where $\frac{1}{n_{\text{eff}}}\frac{\text{d}n_{\text{eff}}}{\text{d}T}$ is the thermo-optic coefficient, which is approximately equal to 8.6 × 10^−6^ K^−1^ for germanium doped silica core optical fiber and $\frac{1}{\Lambda }\frac{\text{d}\Lambda }{\text{d}T}$ is the thermal expansion coefficient of the optical fiber, which is approximately equal to 0.55 × 10^−6^ K^−1^ for silica so that the refractive index change is by far the dominant effect [5]. The order of magnitude of the temperature sensitivity of the Bragg wavelength is 10 pm/°C around 1,550 nm.

The second term in Equation (2) represents the effect of longitudinal strain on an optical fiber. It corresponds to a change in the grating periodicity and the strain-optic induced change in the refractive index [10]. Assuming that the grating is strained in the *z* direction only and that the fiber material follows Hooke's law, the Bragg wavelength shift as a result of the applied strain is defined by:

(4) $$\Delta \lambda_{B}=\lambda_{B}(1-p_{e})\Delta \varepsilon$$

(5) $$p_{e}=\frac{n_{\text{eff}}^{2}}{2}[p_{12}-\upsilon (p_{11}+p_{12})]$$

Substitution of parameters (p_11_ = 0.113, p_12_ = 0.252, ν = 0.16 and n_eff_ = 1.482 [11]) in Equations (4) and (5) give a strain-optic constant p_e_ = 0.21 and an axial strain sensitivity of the Bragg wavelength of 1.2 pm/με around 1,550 nm.

**Figure 2.**
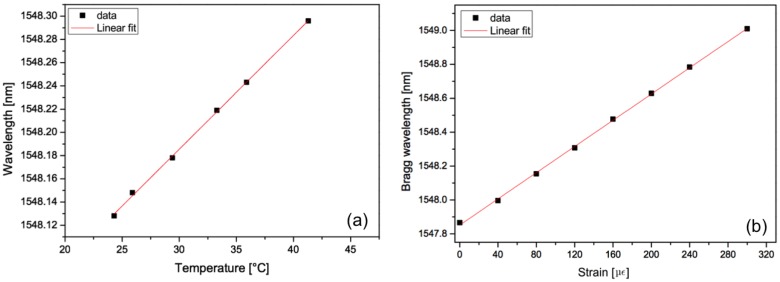
Bragg wavelength shift as a function of a temperature change (**a**) and a mechanical axial strain (**b**).

Figure 2 displays the evolution of the Bragg wavelength shift as a function of temperature (left) and an axial strain change (right). They were respectively obtained thanks to an oven accurate to 0.1 °C and a traction system accurate to 1 με where the optical fiber containing the FBG was fixed between two supports, one fixed and one mobile. The evolutions are linear and without hysteresis, which is exquisite for sensing purposes.

As already mentioned, the main advantage of FBG sensors is that the information about the perturbation is wavelength-encoded. This property makes the sensor self-referencing and independent of fluctuating light levels. The system is therefore immune to source power and connector losses that affect many other types of optical fiber sensors. The very low insertion loss and narrowband wavelength reflection of FBGs offer convenient serial multiplexing along a single-mode optical fiber. There are further advantages of FBGs over conventional electrical strain gauges, such as linearity in response over many orders of magnitude. Furthermore, FBGs can be easily embedded into materials to provide damage detection or internal strain field mapping. FBG sensors are therefore very important components for the development of smart structure technology and for monitoring composite material curing and response.

## 3. Discrimination between Temperature and Strain Effects

Temperature and strain are among external parameters that may affect composite materials. It is naturally interesting to discriminate between both effects. According to Equation (2), a single FBG cannot achieve such discrimination, as both temperature and strain induce a Bragg wavelength shift. Two FBGs exhibiting different temperature and/or strain sensitivities are used in practice. Here is a non-exhaustive list of the main solutions used for temperature-strain discrimination:

- Use of FBGs photo-inscribed in different types of fiber [12,13]: the chemical content of the optical fiber influences the FBG sensitivity. This solution is limited for use in composite materials due to the splice that may weaken the sensor integrity.
- Use of FBGs photo-inscribed at two different wavelengths in a single optical fiber [14]. Table 1 summarizes temperature and strain sensitivities for a standard optical at different wavelengths, taken from [15]:
- Exploitation of the first and second diffraction orders of a single FBG [16]. As in the previous solution, using different wavelengths provides different sensitivities. In both cases, a broadband optical source or different narrowband optical sources are needed, increasing the cost of the demodulation technique.
- Use of specialty optical fibers (polarization maintaining fibers [17,18] or photonic crystal fibers [19,20]) that yield a very well-conditioned system. Here, attention has to be paid on the splicing between specialty and standard fibers as well as on the injection of light. The input state of polarization has to been tightly controlled.
- Processing of the gratings such as encapsulation, decrease of the optical fiber diameter or adjunction of a special coating [21–23]. These operations are time consuming and could weaken the optical fiber.
- Coupling a Fabry-Perot interferometer to an FBG [24]. This solution requires using two different kinds of interrogators.
- Use of hybrid gratings such as a uniform FBG and a long period fiber grating (LPFG). They can be co-located to yield a localized discrimination between temperature and strain [25,26].
- Use of FBGs of different types such as type I, type IA or type IIA gratings [27,28].
- Use of tilted FBGs that couple light both in the core and the cladding and therefore display numerous cladding mode resonances in their transmitted spectrum. Each cladding mode resonance behaves differently in response to strain [29–32].

In the following, we detail some configurations based on Bragg gratings manufactured into standard single-mode optical fibers and compare their relative performances.

### 3.1. FBGs Pair with One of Them Embedded into a Glass Capillary

The simplest way to isolate temperature and strain effects consists in using two FBGs whose one is embedded in a capillary and is therefore immune to strain changes. In the following, we present the results of an experiment conducted on a 2.8 mm thick composite material sample made by stacking together 8 plain-weave layers of carbon reinforcing fibers. Manual stratification was operated with a matrix made of a mix between epoxy resin XB 3585 and hardener XB 3403 (Huntsman, The Woodlands, TX, USA) whose curing cycle is 8 h at 80 °C. Two FBGs were sandwiched between the 4th and 5th layers. One of them was embedded into a glass capillary (500 μm inner diameter and 850 μm outer diameter). The capillary was sealed at both ends to prevent resin intrusion. The final dimensions of the composite sample are 8 cm × 25 cm. The sample was subject to temperature changes in the range between −20 °C and 60 °C (below the polymerization temperature) and to axial strains in the range between 0 and 2000 daN.

**Figure 3.**
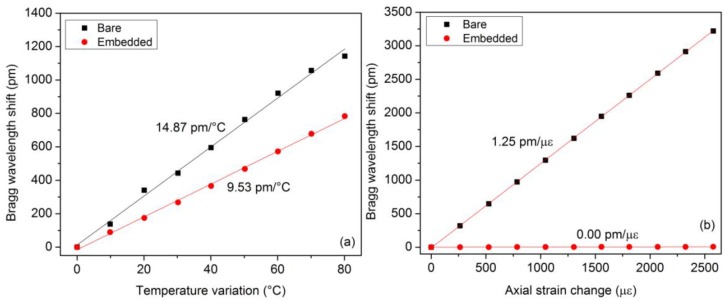
Bragg wavelength shifts as a function of strain and temperature for both FBGs integrated into the composite material sample.

Reflected amplitude spectra were measured with a FiberSensing FS2200 interrogator with a 1 pm wavelength resolution. The Bragg wavelength shift was tracked in both spectra as a function of the external parameter. Figure 3 depicts the obtained evolutions as a function of strain and temperature. One can notice that the FBG embedded into the capillary is totally insensitive to axial strain. Also, the temperature sensitivity of the FBG in direct contact with the composite material is ∼50% higher than the embedded FBG. This results from the fact that this grating undergoes the temperature change supplemented by a thermally-induced mechanical deformation. These results can be expressed using the following expression [33]:

(6) $$\left(\begin{array}{c} \Delta \lambda_{\text{B},\text{bare}}\\ \Delta \lambda_{\text{B},\text{caillary}} \end{array}\right)=\left(\begin{array}{cc} {\text{K}}_{\varepsilon ,\text{bare}} & {\text{K}}_{\text{T},\text{bare}}\\ {\text{K}}_{\varepsilon ,\text{caillary}} & {\text{K}}_{\text{T},\text{caillary}} \end{array}\right)\left(\begin{array}{c} \varepsilon \\ \Delta T \end{array}\right)$$

where the *K* matrix contains the strain and temperature coefficients of both FBGs (bare and embedded into the capillary). Its determinant reflects the decoupling efficiency. The latter increases with the determinant value. According to [34], uncertainties on strain and temperature can be computed from the uncertainty on the Bragg wavelength determination (δλ_b_):

(7) $$\left|\delta \text{T}\right|\leq \frac{\left|K_{\varepsilon ,\text{bare}}\right|\left|\delta \lambda_{B}\right|+\left|K_{\varepsilon ,\text{capillary}}\right|\left|\delta \lambda_{B}\right|}{\left|D\right|}$$

(8) $$\left|\delta \varepsilon \right|\leq \frac{\left|K_{\text{T},\text{bare}}\right|\left|\delta \lambda_{B}\right|+\left|K_{\text{T},\text{capillary}}\right|\left|\delta \lambda_{B}\right|}{\left|D\right|}$$

where D is the determinant of the 2 × 2 matrix.

### 3.2. FBGs Pair Comprising Type I and Type IA FBGs

As defined in [35–37], type I FBGs are standard uniform gratings while type IA FBGs are regenerated. The regeneration process occurs in hydrogen-loaded photosensitive (core co-doped with boron and germanium) optical fiber under prolonged exposure to the UV laser beam. With the accumulation of the laser power, the type I grating is erased and another grating is then reconstructed at a higher wavelength (typically between 5 and 15 nm). The reconstructed grating is called type IA. Its higher wavelength results from an increase of the core refractive index above its mean value. Type IA gratings can be conveniently produced by photo-bleaching the optical fiber with a uniform UV beam (process known as pre-exposition technique) prior to its exposition with the phase mask technique [35].

**Figure 4.**
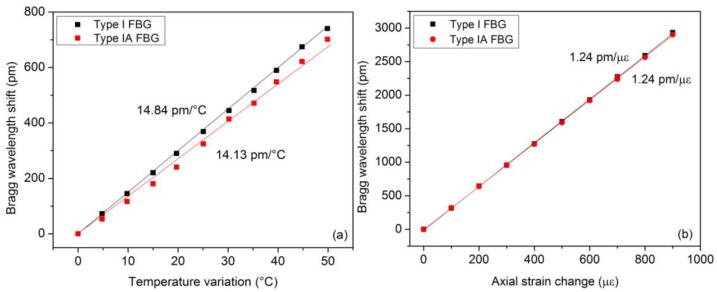
Bragg wavelength shifts of both grating types as a function of strain and temperature.

For the results reported hereafter, both types of FBGs were produced in hydrogen-loaded photosensitive single-mode optical fiber (PHOBBDCDC15 from POFC, Miao-Li County, Taiwan). The writing of the type IA FBG was done in two steps, as explained above. The pre-exposition process under a constant UV power of 80 mW lasted roughly 10 min. Both gratings were 4 mm in length and spaced by only 1 cm to behave as much as possible as a point sensor. Both gratings were annealed at 100 °C during 24 h. The composite material sample was subject to similar solicitations as the ones reported in Section 3.1. Figure 4 depicts the measured wavelength shifts and the corresponding temperature and strain sensitivities. A relationship similar to Equation (8) can also be derived here.

### 3.3. Weakly Tilted FBG

**Figure 5.**
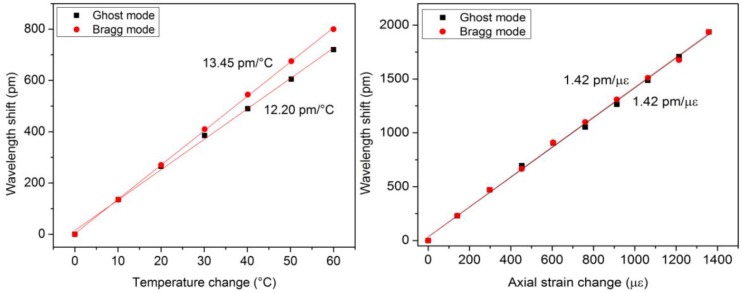
Wavelength shifts as a function of strain and temperature for the Bragg and ghost mode resonances.

As detailed in [29,38,39], tilted FBGs present a refractive index modulation slightly angled with respect to the perpendicular to the optical fiber axis, which couples light in the core and in the cladding. The TFBGs transmitted spectrum contains several tens of cladding mode resonances below the Bragg wavelength. Each one is characterized by its own effective refractive index, inducing a differential axial strain sensitivity, as reported in [40]. In the following, we report results obtained on a 3 mm long 2° TFBG whose transmitted spectrum exhibits two main resonances, the Bragg mode and the ghost mode (resulting from the overlap of low order cladding modes). This grating was embedded in a plain-weave composite material sample in the same way as the one reported in Section 3.1. These two resonances present different temperature sensitivities as observed in the wavelength shifts reported in Figure 5. The axial strain sensitivities are roughly the same.

### 3.4. Comparison of Performances

In this section, we evaluate the performances of the three solutions detailed above. The determinant of the *K* matrix that can be written in the general form *D* = *K_ε,1_K_T,2_*−*K_ε,2_K_T,1_* (where subscripts 1 and 2 refer to the two resonances used for discrimination purpose) has to be different from 0 to ensure the decoupling. Table 2 compares the relative performances of the proposed solutions where the uncertainties were computed according to Equations (9) and (10) using 1 pm for the wavelength resolution of the measurement device. These theoretical uncertainties depend of course on the measurement device.

In [26], it was proposed to estimate the efficiency *E* of the decoupling method based on the following relationship that does not take into account the interrogation system:

(9) $$\text{E}=\frac{\text{D}}{\sqrt{\left(K_{T,1}^{2}+K_{T,2}^{2})(K_{\varepsilon ,1}^{2}+K_{\varepsilon ,2}^{2}\right)}}$$

This formula yields an efficiency of 54% for the first solution based on the capillary, 2.5% and 6% for the other two configurations. Hence, based on this single parameter, the first solution remains the most competitive. However, when taking into account other parameters such as the ease of use, the integration into the composite material sample or the multiplexing capability, using a capillary is for sure less attractive than other configurations. With the increase of the sensor diameter, the glass capillary unfortunately maximizes the distortion of the composite material around its location [7,8]. Hence, there is not a unique solution to the discrimination issue between temperature and axial strain effects. And the best solution is certainly not the one that maximizes the decoupling efficiency. One has to find the good trade-off between metrological and physical performances with respect to the target application and to the composite material composition and geometry.

## 4. Demodulation Techniques for Embedded FBGs for SHM and Curing Cycle Monitoring

Different interrogation techniques are available to demodulate the reflected/transmitted spectrum of FBGs embedded into composite materials. Their choice arises from considerations mainly related to their resolution, sensitivity, accuracy, dynamic range, cost and performances in (quasi-)static (<10 Hz) or dynamic (>10 Hz) measurements. In this section, we first review the main techniques that are used to demodulate FBGs embedded into composite materials when they are subject to external perturbations. We then focus on strategies developed to measure residual stress (most often small values with a strong temperature cross-dependence) arising during the fabrication process. Techniques are classified according to their demodulation mechanism, either based on wavelength detection or based on reflectometry.

### 4.1. Techniques Based on Wavelength Detection

**Figure 6.**
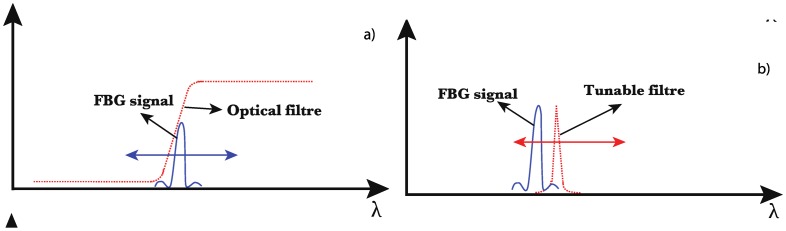
Operating principle of the edge filter (**a**) and tunable filter (**b**) techniques.

Another distinction can be made between active and passive systems, depending on the use of a wavelength sweeping mechanism. Passive demodulation techniques make use of an optical filter whose transfer function depends on the wavelength and converts the Bragg wavelength shift into an amplitude change [41]. Such a filter presents a bandwidth several times higher than the one of the FBG, as sketched in Figure 6a. To obtain an adequate filter, one can use a tilted fiber Bragg grating operating in radiation mode [42], a chirped fiber Bragg grating [43] or a long period fiber grating [44], as they all present a smooth evolution. The use of intensity referencing is necessary as the light intensity may fluctuate with time. This could occur due to unwanted power fluctuations of the light source, disturbances in the light-guiding path or dependency of the light source intensity on the wavelength. Hence, robust demodulation techniques based on an edge filter use a ratiometric scheme to make them independent of intensity fluctuations. In this case, the input light is split into two paths, one passing through the wavelength dependent filter and one used as the reference arm. The wavelength is accurately determined from the ratio between the powers measured at both arms [41]. Such a system is robust and can be set-up at a relatively low cost. However, it can only resolve a single FBG.

Active systems make use of a tunable filter (with a bandwidth narrower than the one of the FBG, as illustrated in Figure 6b) missing fig to scan the FBG amplitude spectrum, which is compatible with wavelength-division multiplexing (WDM) [45]. With similar performances in terms of wavelength resolution and scanning speed, a tunable laser source and a photodetector can be combined to demodulate the FBGs amplitude spectrum. Commercially available solutions based on such an implementation typically offer a 1 pm wavelength resolution and a frequency repetition that can reach 1 kHz for the most advanced systems. The use of an optical spectrum analyzer is another possibility of demodulation. It is essentially deserved to lab experiments, as such a system is slow (several seconds to scan a wavelength range of a few tens of nanometers), cumbersome and expensive.

WDM techniques can be used to demodulate several tens of FBGs [46]. The operating ranges of the optical source and the detector limit the number of sensors that can be cascaded along a single optical fiber, considering that a sufficiently wide channel (2–3 nm) should be left for each FBG to avoid crosstalk.

### 4.2. Reflectometric Demodulation Techniques

To overcome the limitations of WDM systems in terms of bandwidth, other techniques such as time-division multiplexing (TDM), spatial-division multiplexing (SDM) and their combination have been used [47–49]. It has been recently demonstrated that a wavelength-tunable optical time domain reflectometer (OTDR) can be used to interrogate a cascade of identical FBGs (*i.e.*, same Bragg wavelengths under identical operating conditions and reflectance below 10%) [50–54]. An OTDR launches optical pulses in the fiber under test and detects the backscattered and reflected signals. As it shows the magnitude and location of any losses and reflections along the fiber length, detection and localization of tens of sensors along a unique optical fiber is achievable. However, a standard commercial OTDR is not directly suitable to demodulate FBGs sensors: its internal optical source is too broad (several tens of nanometers) and not wavelength tunable so that it cannot track the wavelength shift of perturbed FBGs. Because of these two limitations, it is required to modify a classical OTDR into a wavelength-tunable one with a spectral width much narrower (typically a few tens of picometers) [52]. In [53], an external tunable laser source and some active components (e.g., pulse generator, acousto-optic modulator) are used for this purpose. The method presented [54] is a cost-effective and passive solution based on the use of an appropriately designed edge filter and a classical OTDR. However, techniques based on OTDRs can only be used for static measurements (traces have to be accumulated over a few seconds at least to filter out the measurement noise). Also, several meters of fiber have to be left between consecutive gratings due to the limited spatial resolution. OTDRs are thus marginally used for strain measurements in composite materials [55].

OFDRs (optical frequency domain reflectometers) are much more interesting in practice, offering both a high spatial resolution and a fast scanning rate [56–58]. A coherent OFDR exploits a frequency-modulated continuous wave interference produced by a tunable laser source whose frequency can be swept continuously in time without mode hopping and an optical interferometer (Michelson) comprising a reference path and a measurement path. The fiber under test is connected to the measurement path while the reference path terminated by a mirror is used as local oscillator. Interferences between the two paths are electrically detected and a Fourier transform allows the visualization of beat frequencies. If the optical frequency of the tunable source is modulated at a constant rate, beat frequencies are proportional to the optical path differences between the reflections in the fiber under test and the reference path. OFDRs can provide millimetric spatial resolution over a fiber length of a few hundreds of meters.

**Figure 7.**
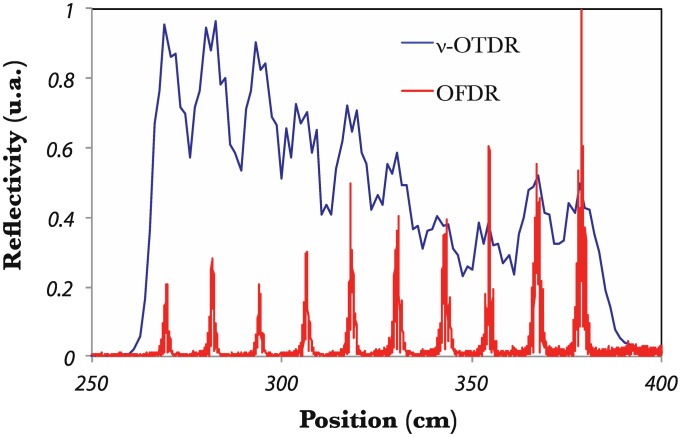
OFDR trace for 10 identical FBGs cascaded in an optical fiber embedded into a composite material fabric and corresponding photon-counting OTDR trace.

In [59], the OFDR developed in [58] was used to demonstrate its potential for the demodulation of closely spaced FBGs embedded into a composite material fabric. 10 FBGs (3 mm in length) were embedded with a spacing between them of ∼10 cm. Figure 7 depicts the corresponding trace in the frequency domain converted into absolute position. A comparison with the same cascade measured by a photon-counting OTDR (high resolution OTDR, as explained in [60]) is also displayed. One can readily see the superior spatial resolution offered by the coherent OFDR. To recover the reflection spectrum of each FBG in the array, the extracted peak by the way of a band-pass filter is inversely Fourier transform. As a result, Bragg wavelength shifts can be recorded for all the gratings.

Other demonstrations of the use of an OFDR for SHM in composite materials can be found in [61,62].

### 4.3. Residual Stress Measurements during the Curing Process

Residual strains appear during the fabrication process of composite materials and may drastically weaken the final structure, increasing the risk of sudden breakage. During the heating process followed to ensure the proper polymerization of the matrix, both the reinforcing fibers and the matrix undergo thermally- and chemically-induced dimensional changes that are responsible for the appearance of residual strains [63–65]. They find their origin either inside the layer stacking (intrinsic residual strains) or at the interface between the piece and the mold (extrinsic residual strains). In 2006, Wisnom *et al.* have identified three main causes for the appearance of residual strain [66]: thermal expansion of the resin [67], volume shrinkage of the resin [63,68] and differential thermal expansions between the mold and the sample [69].

There are numerous techniques to measure residual strains that can be classified into destructive techniques (hole-drilling method [70], layer removal [71]) and non-destructive techniques (X-ray diffraction [72,73], bending measurement [74], ultrasound waves [75], …). FBGs sensors of course belong to the non-destructive category. They have been used to follow the curing cycle [76,77], the resin polymerization process [78,79] and the formation of residual strain [80]. Studies have first focused on the measurement of strain along the fiber axis, neglecting the influence of transverse strains. In [81], Colpo *et al.* have used a 24 mm long FBG interrogated by an optical low coherence reflectometer (OLCR) to measure the axial strain distribution of the resin during the polymerization process. Eum *et al.* have reported the use of a 10 cm long FBG interrogated by an OFDR to follow the residual stress appearing during a vacuum-assisted resin transfer molding process [82]. In [83], a Brillouin optical time domain analysis was used for a similar purpose. In all of these works, the focus was only made on the measurement of longitudinal strains. Transverse strains are mentioned in [84] without deep investigations on their value. Their precise measurement during the fabrication process is of high importance because they are associated to the most fragile direction and can be at the origin of delamination.

FBGs are intrinsically sensitive to transverse strain. However, the obtained effect is not a pure wavelength shift but a broadening/splitting of the reflection band since transverse strains create birefringence. Birefringence in optical fiber is due to the presence of asymmetries in the cross section. In FBGs, it is commonly induced during the writing process in addition to the intrinsic fiber birefringence [85]. While this induced birefringence is hardly perceived in the grating amplitude response, it leads to significant polarization-dependent loss (PDL) [86]. Birefringence (Δn) is defined as the difference in refractive index between two orthogonal polarization modes (or eigenmodes) labeled x and y modes. The refractive indices associated to these modes are defined by n_eff,x_ = n_eff_ + Δn/2 and n_eff,y_ = n_eff_−Δn/2, where is the core effective refractive index without birefringence. Due to Δn, the x and y modes undergo different couplings through the grating. Therefore, the total FBG spectrum is the combination of the x and y mode signals. The wavelength spacing between both modes is defined by 2ΛΔn [87]. Hence, for increasing birefringence values, a broadening of the reflection band is first obtained before both polarization modes become well separated [88–92]. At this stage, the transverse strain value can be estimated from the measurement of the wavelength spacing between both peaks. This can be achieved for transverse force values exceeding a few tens of N [93,94]. To overcome this limitation and allow for residual transverse stress measurement, different configurations have been reported:

- FBGs photo-inscribed in polarization maintaining (bow-tie) fibers [95] with an amplitude spectrum displaying two peaks;
- FBGs manufactured in highly-birefringent photonic crystal fibers [96,97], further improving the resolution provided by standard polarization maintaining fibers thanks to an increase of the wavelength spacing between both peaks and a temperature insensitivity. Polarization maintaining fibers exhibit a strong orientation-dependent sensitivity. Accurate measurements can only be achieved after a correct orientation of the fiber;
- Standard FBGs with measurement of their spectral broadening for small transverse strain values [98];
- Standard FBGs with exploitation of their polarization dependent loss spectrum. It was shown in [99,100] that the PDL spectrum contains much more information than the amplitude spectrum, which can be accurately used to detect transverse strain build-up due to matrix polymerization. A differential transverse strain of 60 με was measured for cross-ply laminates.

Additional considerations on multi-axis strain sensing can be found in [101,102]. The state-of-the-art review again confirms that there is no universal solution to demodulate FBGs sensors during and after the curing process. Table 3 summarizes the metrological performances of the solutions available to follow the curing process. With the use of dedicated interrogators for FBGs sensors, they are quite competitive in terms of wavelength resolution and cost, the main difference being related to the use or not of polarized light.

### 4.4. Non-Uniform Strain Sensing

Techniques described in Sections 4.1 and 4.2 properly work when the strain distribution is uniform over the grating length. However, non-uniform longitudinal strains tend to introduce an uncontrolled chirp in the modulation of the refractive index, which in turn strongly affects the amplitude spectrum and prevents the correct operation of standard demodulation techniques. Non-uniform strains can be generated from damages such as cracks, debonding, delaminations [103–105], non-uniform shrinkage [81] or bending [106]. Resulting spectra may be relatively simple when the delamination is parallel to the FBG axis or much more complicated when non-uniform birefringence effects come into play.

Different studies have focused on the FBG amplitude spectral deformations in such conditions, as reviewed in [7]. Other works have demonstrated advanced techniques to demodulate FBG sensors or to evaluate the strain distribution in the presence of non-uniform strain along their physical length. A non-exhaustive list yields the following strategies:

- Exploitation of both the amplitude and phase spectra of FBGs through the use of the Fourier transform, yielding a spatial resolution of about 1 mm [107];
- Use of the optical low coherence reflectometry (OLCR) combined with uniform FBGs, yielding a spatial resolution better than 1 mm [108];
- Use of digital image correlation technique together with FBGs [109];
- Exploitation of the micro-computer tomography to investigate the internal structure of embedded optical fibers [110].

## 5. Connecting Embedded Fibers with the Surroundings: Towards Industrialization of the Process

In this last section, we will review a crucial issue related to embedded optical fiber sensors: the entry point, which is very fragile and requires a lot of caution especially when working with a molding process. In 1999, Green *et al.* overviewed the main approaches to overcome this limitation [111]. Since then, numerous developments and progress have been made, as solving the egress/ingress issue is a mandatory step towards the industrialization of the SHM process with embedded optical fibers in composite materials. The proposed techniques can be classified in four classes.

### 5.1. Bare Optical Fibers

**Figure 8.**
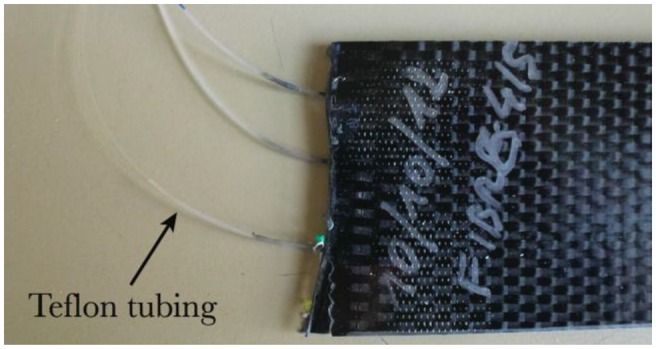
Protective loose tube in Teflon around the optical fiber.

It is a straightforward method consisting in adding a loose tube protection around the optical fiber at the edge of the composite materials sample. The tube can be in polyvinylidene fluoride (PVDF) or polytetrafluoroethylene (PTFE, also known as Teflon) [99,112]. This technique is useful for lab tests but it is not realistic for industrial applications, as it prevents the proper cutting and polishing of the composite material edges at the optical fiber location. Figure 8 illustrates this kind of connection.

### 5.2. Surface-Mounted Connectors

**Figure 9.**
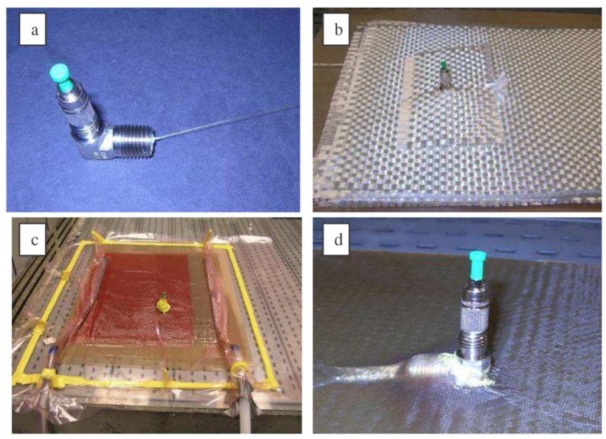
Main steps towards the integration of surface-mounted connector, from [113].

This solution consists in using commercially available standard connectors for optical fibers and mounting them from the top of the composite material sample, as depicted in Figure 9 [113,114]. It yields a robust connection and allows an easy cutting and polishing of the material. However, the fabrication is somehow complex as the optical fiber has to cross different layers. The optical fiber curvature should also be tightly controlled to avoid important power loss.

### 5.3. Edge Connectors

**Figure 10.**
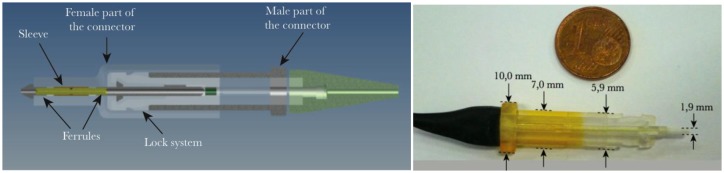
Edge connector made with the SLA technique (sketch and realization from [119]).

This solution makes use of specially designed connectors, most often miniaturized with respect to standard telecommunication-grade connectors. The fabrication is simplified with respect to surface-mounted connectors while the robustness is equivalent [115–117]. The proper cutting/polishing issue was solved with a miniaturized magnetic connector [118] or with a polymer-based connector realized with a stereolithography apparatus (SLA) [119]. The obtained connector is depicted in Figure 10.

### 5.4. Free-Space Connections

Bare fiber pigtails are very fragile, edge connectors inhibit connections to integrated panels, and surface connectors result in mechanical protrusions and breakage points. These problems may be avoided by using free-space optics for signal input/output. The embedded fiber sensors must be optically prepared to accept a free-space optical signal from an optical interrogator unit positioned anywhere from a few mm to a few meters away. One way to do this is shown in [98], where a panel with an embedded fiber sensor is cut, exposing a cross-section of the fiber, which was mechanically polished with sandpaper, allowing light from the fiber sensor to be detected with an external receiver. This output-only free space coupler showed 3 dB loss, from the roughness of the fiber face. More desirable is to interrogate the sensor from the surface, that is, from a direction perpendicular to the fiber. References [120–122] demonstrated this for both input and output to the fiber sensor by forming thin slits through the panel at 45 degrees, then polishing the exposed fiber cross-sections with fine sandpaper and lapping film to produce reflective turning mirrors. In [120–122], the embedded fiber sensor was fused to multimode optical fiber sections that provided the optical input coupling mirrors, while the output coupling mirrors are directly polished onto the single mode fiber. Total optical losses including transmission losses from the multimode sections to the singlemode sensor sections, and input and output free space coupling losses, were ∼ 20 dB. Despite these losses, this configuration was still sufficient to obtain excellent signal quality and measurement of the Bragg resonance. Experimental data confirmed that optically transduced strain data matched that of electrical strain sensors placed on the panel, demonstrating the viability of the use of 45 degree polished mirrors to provid;e free-space interrogation of embedded fiber sensors.

## 6. Conclusions

Taking into account their miniaturized dimensions and relative robustness, fiber Bragg gratings are ideal sensors for integration in composite materials. They can be used for structural health monitoring but also to reveal the residual stress build-up during the fabrication process. Despite their specific advantages, the proper use of FBGs requires skills and practice. Numerous solutions are disseminated in the scientific literature since many years now. This review article has focused on three major issues arising from the use of FBGs embedded into composite material samples: the discrimination between temperature and strain effects, the amplitude spectrum demodulation comprising the measurement of residual stress and the connection interface between the embedded sensors and the surroundings. These challenges were deeply investigated and while most of the issues were tackled, there is still some room for progress, especially for the connection aspect and for the curing cycle monitoring. The former is essential for an industrialization of the process. Two antagonist approaches appear very competitive, free-space connection, on the one hand, and designing a dedicated connector, on the other hand. The curing cycle monitoring has not yet revealed its full potential. Numerical simulations made with a finite element mode solver can surely bring insight to fully understand and interpret the amplitude spectrum modifications resulting from the curing process. Finally, thanks to their important development during the past few years, polymer optical fiber gratings now appear mature for use in composite materials, providing that the temperature is kept below 60–70 °C [123,124]. The polymer nature of the optical fiber makes it hydrophilic, which can be advantageously used during the cure cycle.

## Figures and Tables

**Table 1.** FBG strain and temperature sensitivities at different wavelengths.

**Wavelength (nm)**	**Strain Sensitivity (pm/με)**	**Temperature Sensitivity (pm/°C)**
830	0.64	6.8
1300	1.00	10.0
1550	1.20	13.0

**Table 2.** Comparison of performances between the three investigated solutions for temperature-strain discrimination.

**Method**	**Matrix K**	**Determinant (pm**^2^**/με °C)**	**Temperature Uncertainty (°C)**	**Strain Uncertainty (με)**
Bare + Capillary	**$\left[\begin{array}{cc} 0 & 9.53\\ 1.25 & 14.87 \end{array}\right]$**	−11.912	0.1	2
Type I + type IA	**$\left[\begin{array}{cc} 1.24 & 14.84\\ 1.24 & 14.13 \end{array}\right]$**	−0.880	2.8	33
Ghost + Bragg	**$\left[\begin{array}{cc} 1.42 & 13.45\\ 1.42 & 12.20 \end{array}\right]$**	−1.775	1.6	14

**Table 3.** Performances of techniques used for strain build-up measurement during the curing process.

**Technique**	**Sensing Modalities**	**Requirements**
Standard FBGs (Amplitude spectrum)	- Sensitive to axial and transverse strains - Sensitive to temperature	- Need for temperature compensation for axial strain measurements (Cf. section 3)
Standard FBGs (PDL spectrum)	- Sensitive to axial and transverse strains - Temperature compensation	- Use of polarized light
Hi-Bi FBGs (Amplitude spectrum)	- Sensitive to axial and transverse strains	- Use of depolarized light - Need for proper fiber orientation - Need for temperature compensation
Hi-Bi PCF FBGs (Amplitude spectrum)	- Sensitive to axial and transverse strains - Immune to temperature changes	- Use of depolarized light - Need for proper fiber orientation - Need for controlled splices
